# Mental Health Disorders in Ultra Endurance Athletes per ICD-11 Classifications: A Review of an Overlooked Community in Sports Psychiatry

**DOI:** 10.3390/sports11030052

**Published:** 2023-02-23

**Authors:** Jill Colangelo, Alexander Smith, Ana Buadze, Nicola Keay, Michael Liebrenz

**Affiliations:** 1 Department of Forensic Psychiatry, University of Bern, 3012 Bern, Switzerland; 2 Department of Psychiatry, Psychotherapy and Psychosomatics, Psychiatric Hospital, University of Zurich, 8008 Zurich, Switzerland; 3 Division of Medicine, University College London, London WC1E 6DE, UK

**Keywords:** ultra-endurance athletes, sports psychiatry, mental disorders, training volume, ICD-11, endocrine dysfunction

## Abstract

Introduction: Although research suggests that exercise benefits mental health, psychiatric disorders have been acknowledged in the ultra-endurance-athlete population. At present, the mental-health consequences of high-volume training associated with ultra-endurance sports are not well understood. Methods: We conducted a narrative review summarizing primary observations about mental disorders per ICD-11 criteria in ultra-endurance athletes using a keyword search in Scopus and PubMed. Results: We identified 25 papers discussing ICD-11-classified psychiatric disorders such as depression, anxiety, eating disorders, attention-deficit/hyperactivity disorder, and schizophrenia in ultra-endurance athletes. Discussion: Although evidence is limited, available papers indicate that there is a sizable incidence of mental-health issues and composite psychopathological vulnerabilities in this community. We contend that ultra-endurance athletes may represent a different, though similar, demographic than elite and/or professional athletes, as they often engage in high-volume training with similarly high motivation. This can have regulatory implications, which we also highlight. Conclusion: Mental illness in ultra-endurance athletes is an underrepresented topic in sports medicine, though psychiatric disorders may be especially prevalent in this population. Further inquiry is necessary to inform athletes and healthcare practitioners about the possible mental-health implications associated with participation in ultra-endurance sports.

## 1. Introduction

There is evidence of increased psychiatric disorders in the ultra-endurance-athlete (UEA) population, despite the known mental-health benefits of exercise [1]. Anecdotally, it could be argued that mental illness is a commonly recognized feature of the UEA community [2]. Mental-health improvements have been noted in individuals exercising at moderate yet consistent volumes [3,4,5,6]. Resistance exercise has been known to improve brain structure, function, and connectivity through regulation of neurotrophic factors [7]. Specifically, brain-derived neurotrophic factor (BDNP) may have antidepressant effects [8], suggesting some measure of exercise can help prevent psychiatric issues [9]. However, discussion of UEA training volumes indicates that they are exercising beyond moderation, as high-volume training is a necessary characteristic of participation in ultra-endurance sports (UESs) like ultramarathon, Ironman triathlon, mountain sports, endurance cycling, or endurance swimming [10]. Although there is no specific definition of UES races, they typically span durations over six hours or over several days and appropriate training includes continual addition of volume [11]. Accordingly, our criteria for UEAs follows the classifications proposed by Scheer and Knechtle and Nicolaidis [10,11]—that is, athletes training for ultramarathons of any distance over 42 km or other ultra-distance events lasting six hours or more. Investigations show that psychiatric issues may result from multiple hours of competition in an intra-race situation or from wider circumstantial paradigms, like the COVID-19 pandemic [12,13]. Although they provide useful insights, we believe that these studies do not fully capture the intrinsic psychological profile of the athlete, independent of external and contextual factors. In the limited investigations available, high-volume training is associated with a decrease in athlete well-being and a decline in mood [14,15,16,17,18].

Despite indications of psychiatric issues and the publication of a review article introducing this topic [1], there have been no formalized efforts to collate extant information to create an official mental-health statement about this community. This contrasts with other sporting domains, where researchers have developed guidelines for both high-performance athletes [19] and elite athletes [20]. Correspondingly, there are minimal regulatory interventions around mental health in UEAs, particularly as UESs are heterogeneous and involve multiple stakeholders internationally. This is distinct from more established sporting disciplines that may be subject to greater scrutiny and emphasize mental health initiatives, such as those addressed by the formulation of organizations like the International Olympic Committee Mental Health Working Group [21].

A UEA may be elite or recreational, though they might be training at similar levels of volume and intensity and can be driven by similar intensity of motivation as high-performance athletes [22,23]. Recreational athletes may experience additional pressures associated with balancing heavy training loads with professional and familial responsibilities without the benefit of a support team [24]. Additionally, it is acknowledged within the community that UEAs often drift from one sport to another, as their physical capabilities, motivations, and interests draw them to complementary challenges. First-hand opinions support this; for instance, the Ironman world-champion triathlete, Sika Henry, has observed these patterns [25].

To the authors’ knowledge, detailed research into these considerations is limited and comprehensive evidence on the prevalence of mental disorders in UEAs is scarce. Moreover, it is not well understood whether there are athletes that gravitate toward UESs specifically with the intention of ameliorating preexisting psychiatric symptoms or whether participation in UESs incurs neuropsychological change such that mental disorder develops [1]. There has been broad media coverage of UEAs who use participation in their sport to manage existing symptoms related to post-traumatic stress disorder (PTSD), attention-deficit/hyperactivity disorder (ADHD), autism-spectrum disorder (ASD), and alcohol-use disorder [26,27,28,29,30]. Alternatively, preliminary neurological work into UEAs suggests that high-volume training affects neurotransmitter balance, contributing to risk factors for mental-health issues [31]. Resulting 5-HTTPLR polymorphism may be correlated with severe depression and has links with increased amygdala activation in situations of anger [31,32]. Moreover, the COMT val^158^ met polymorphism critical to the regulation of dopamine may also be prevalent in UEAs, causing athletes to be more prone to novelty-seeking behavior, which has been associated with bipolar disorder, ADHD, psychosis, substance-use disorders, and suicide [33]. Given the forensic–psychiatric implications of potentially increased emotional reactivity, this lack of information is again concerning [34]. More generally, both onset possibilities would create similar, though unique, considerations in the UEA community requiring additional education and possible tailored interventions. 

To address these knowledge gaps, we performed a narrative review to synthesize current literature about mental disorder in UEAs. We summarized our results and thematized psychiatric symptoms per diagnostic classifications from the ICD-11 (as distinct from the DSM-V) and focused our search on the World Health Organization’s (WHO) list of the most prevalent mental disorders [35,36,37]. Based on the evidence we collated, we discuss our findings and their relevance for UEAs in the broader framework of sports medicine and sports psychiatry. 

## 2. Methodology

We searched two academic databases in November 2022, Scopus and PubMed, for scholarly materials on UEAs and mental disorders as classified within ICD-11 diagnostic domains [35]. No timeframe parameters for published materials were adopted in our methodology. The search was conducted using the terms “ultra-endurance athlete,” “ultrarunner,” “ultramarathoner,” “endurance cyclist,” “triathlete,” “mental disorder,” and “mental illness,” along with terms from the WHO’s list of the most common mental disorders: “depression,” “anxiety,” “bipolar,” “post-traumatic stress disorder,” “schizophrenia,” “eating disorder,” “attention-deficit/hyperactivity disorder,” and “autism” [36]. Subsequently, we manually added pertinent articles from applicable reference lists. Following this, we removed papers that did not report primary empirical observations (e.g., review articles), duplicates, and those not written in the English language. Furthermore, we excluded samples that did not fulfill our definition of ultra-endurance sports as delineated above and where this could not be definitively determined from cohort descriptions. In addition, we omitted papers designed to reveal mental-disorder concerns exclusively due to other contextual factors, like the COVID-19 pandemic, or psychiatric and psychological issues specifically resulting from in-competition conditions.

## 3. Results

Per our search criteria, we identified 25 papers that show evidence of mental disorder in UEAs. Studies included in our review are summarized in Table 1 in chronological order with an outline of their methods, instrumentation, and results.

We thematized symptomatology in these reviewed papers per ICD-11 diagnostic domains for readability and structure. Aside from those specifically mentioned below, our search methodology did not return evidence for other disorders classified within ICD-11.

### 3.1. Mood Disorders 

#### 3.1.1. Depression (6A7Z) 

A series of investigations into 400 swimmers found that statistically significant (*p* < 0.005) increases in depression, as measured by the POMS over a five-month period, correlated with the highest-volume training block [14]. The same study was repeated over a 10-day period of increased training where sizable increases (*p* < 0.005) occurred in the ratings of exercise intensity, muscle soreness, depression, anger, fatigue, and global mood disturbance, along with a reduction in general sense of well-being [15]. Triathletes participating in a race had been diagnosed with depression (21%) and were in treatment for mood disorder (12%), though it did not affect their mood during the competition [40]. Ultramarathoners also showed evidence of depression risk via PHQ-2 (20%) [50]. A male triathlete was treated for anorexia nervosa and depression, showed symptoms of psychosis, and was later formally diagnosed with schizophrenia [41]. A study of high-performance athletes noted that those with EXD had a higher likelihood of depression (*t*(121) = 4.944, *p* < 0.001) and childhood trauma (*t*(121) = 2.297, *p* = 0.024) [59].

#### 3.1.2. Anxiety or Fear-Related Disorders (6B0Z)

In triathletes, there was a significant relationship (*r* = 0.32, *p* < 0.001) between anxiety and volume of training as well as between training volume and perceived fatigue (*r* = 0.30, *p* < 0.001) [18]. Research into ultra-endurance runners, cyclists, and paddlers found that paddlers experienced the most anxiety/panic (*χ*2 = 7.91, *p* < 0.01) and also had higher self-loathing scores than cyclists (*F*(2) = 6.91, *p* < 0.01). The most anxiety/panic symptoms were reported by the female-only paddler group (*χ*2(1) = 10.27, *p* < 0.001) [42]. A study using genotyping in triathletes identified that anxiety disorder was a risk for 44.3% of total participants and 57% of the inferior-performance (IP) group. The IP group exhibited higher frequency of polymorphisms for 5-HTTLPR, which is associated with increased anxiety, and 5HT1AR-1019C > G, which is associated with anxiety disorder [47].

#### 3.1.3. Feeding or Eating Disorders (6B8Z), Other Specified Feeding or Eating Disorders (6B8Y)

Seven of the reviewed papers showed evidence of ED in triathletes, endurance cyclists, and ultrarunners. In a sample of male cyclists and triathletes, the total score for EXD was positively correlated with ED risk as measured by EDE-Q (*r* = 0.41, *p* < 0.05) [52]. The same study included biomarkers, finding that subscales of EXDs were negatively correlated with fasting blood glucose (withdrawal *r* = −0.31 and tolerance *r* = −0.32, *p* < 0.05) [52]. The subscale of “intention effect” was negatively correlated with the ratio between testosterone and cortisol *(r* = −0.29, *p* < 0.05) and positively correlated with the ratio between cortisol and insulin (*r* = 0.33, *p* < 0.05) [52]. 

A study of triathletes assessed with EAT-26 observed that 28% of females and 11% of males were at risk for ED [38]. Additional findings included discrepancies in perceived BMI vs. actual BMI, perceived body shape vs. actual body shape, and evidence of undereating in 74.5% of males and 73.9% of females [38]. Dissatisfaction with BMI was noted in 100% of the triathletes [38]. Another investigation into triathletes discovered that 12.3% of the population was at risk for ED using EAT-26 [39].

In a cohort of amateur endurance athletes, the prevalence of ED as measured by EDDS and food addiction as measured by YFAS was found to be 6.25% and 6.5%, respectively [53]. In the same sample, there was a strong statistically significant relationship between FA and EXD (*X*^2^(1) = 15.117, *p* < 0.001, *n* = 1022) [53]. An evaluation of male cyclists utilizing the EDS and DT found that 1.18% were at risk and 10.59% were symptomatic for exercise dependence secondary to ED [48]. Research adopting the TFEQ, the Kessler 6 Scale, and EDS in Ironman triathletes created five profiles based on symptom expression and presence of EXD [46]. In particular, those athletes found to be at risk demonstrated disordered and maladaptive eating patterns [46]. Furthermore, one case study of a male triathlete and one autoethnographic case report of a female triathlete described experiences with anorexia nervosa [41,45].

Research examining ultra-endurance runners, cyclists, and paddlers found their risk for ED to be 12%, 14%, and 18%, respectively. This work also contained evidence of an association between self-loathing and ED symptoms for the entire group (*F*(1) = 4.83; *p* < 0.05) and for females specifically (*F*(1) = 9.30, *p* < 0.001) [42]. Risk for ED was found to be 19.7% in male cyclists using EAT-26 and SEDAC [44].

Risk of ED was high in 11% of male cyclists assessed using the SNKQ and BEDA-Q and negatively correlated with their understanding of nutrition (*r* = −0.55, *p* = 0.006) [56]. In a separate paper, EAT-26 was used to find 80% of female cyclists at risk for ED [57]. Separately, ultramarathoners were assessed with a self-developed survey consisting of questions from the Female Athlete Triad Screening Questionnaire and the Eating Disorder Examination Questionnaire. In this article, risk of ED was found in 44.5% of male and 63.5% of female ultramarathoners, with evidence of BSI found in 37.5% of females and 20.5% of males using DXA [59].

#### 3.1.4. Schizophrenia or Other Primary Psychotic Disorders (6A20.Z)

One study described a male triathlete with both ED and evidence of psychosis [41].

#### 3.1.5. Attention Deficit Hyperactivity Disorder, Presentation Unspecified (6A05.Z)

One investigation found that athletes with EXD had higher likelihood of depression [*t*(121) = 4.944, *p* < 0.001], ADHD [*t*(121) = 2.915, *p* = 0.004], and childhood trauma [*t*(121) = 2.297, *p* = 0.024] [58]. 

#### 3.1.6. Harmful Pattern of Use of Alcohol, Unspecified (6C40.1Z)

Using genotyping in triathletes, researchers noted the presence of NK1R r56715729 polymorphism which is associated with stress homeostasis, alcohol dependence and alcohol abuse [47]. In a longitudinal examination of EXD in amateur endurance cyclists, increased alcohol use was observed in 14.8% of participants [55].

## 4. Discussion

### 4.1. Current Literature on Mental Illness in UEAs

Our results illustrate that UEAs may have various psychiatric issues and face substantial vulnerabilities. Of the twenty-five studies reviewed, ED is evident in fifteen, depression in nine, anxiety in five, alcohol use in two, psychosis in one and ADHD in one. Given the heterogeneity of the UEA community, it is possible that the rates of psychiatric disorders illustrated in these papers do not fully represent the broader prevalence in this population; we believe UEAs constitute an overlooked group in sports psychiatry literature. This is demonstrated by the fact that most of the included studies do not emphasize etiological determinants of mental illness or focus on the trajectory of prior or existing morbidities and how they may be influenced by participation in UES.

Furthermore, based on our methodology, the reviewed articles do not show several psychiatric disorders known to exist anecdotally in this population including, but not limited to, PTSD, ASD, and bipolar disorder [26,27,28,29,30], as we previously outlined. Equally, suicide risk is not discussed in the literature, though personal accounts from UEAs who have psychiatric disorders note that depression and suicidal ideation are intertwined with training for long distance events [60,61]. The associations between extreme sleep patterns in-competition and mental health disorders after the race are also missing from extant literature in our review, but these onset possibilities have been identified in other contexts [62].

As mental health and physical health are inextricably linked [63], the articles in our review offer corresponding evidence of psychological wellbeing that research on the physiological health of UEAs has been often unable to provide. Prior studies have posited that UEAs were “less unwell” due to lower nonattendance at work and school and hypothesized that ultrarunners are healthier by virtue of their capability to train for and complete an ultramarathon [23,64]. It is likely that stigma has influenced perceptions around this population; it has been argued that since UEAs appear to be healthy, they are less likely to be assessed for disorders that can lead to serious conditions [1,47]. For the authors, we believe that with more investigations into the etiology of mental disorders in UEAs, health care practitioners could be encouraged to recognize and attempt to treat this potentially overlooked population.

### 4.2. Diagnostic Considerations and Awareness of EXD/Exercise Dependence, Overtraining Syndrome, and ED

Reviewed evidence indicates a need for increased awareness of the risk of developing EXD/exercise dependence, which is positively correlated with large volumes of exercise and can entail serious health complications [65,66]. Although literature in sports medicine often emphasizes the risk for EXD in elite-level athletes, any athlete who is compelled to exercise irrespective of physical- and/or mental-health considerations could potentially develop a dependence [65]. EXD appeared in nine of the 25 studies, though it is not currently included as a specific category in the ICD-11 or DSM-V [35,37]. The addiction-component model, which utilizes criteria for addiction that appears in both behavioral and problematic substance use, has been adopted to diagnose exercise addiction in female cyclists [67]. Since gambling, a behavioral addiction also diagnosed with the addiction-component model, was added to the ICD-11 in 2022 [68], it is hoped that exercise may be included as well. This is particularly important since the co-occurrence of substance addictions and exercise addiction is generally not well understood and the complexities of treating both has been underlined [69]. 

The lack of EXD inclusion in these manuals simultaneously entails an absence of diagnostic criteria or specific protocols for treatment [70]. In the authors’ opinion, treatment options should be tailored among those at risk for EXD given that it often occurs both with and without concomitant ED [69]. Many UEAs could be at risk for co-morbid diagnoses [69], though our review of the literature suggests considerable differences between athletes with primary vs. secondary EXD [49,70]. Although EXD would be classified as a psychiatric disorder were it to be included in a diagnostic manual, it describes the use of exercise to elicit a psychological effect, such as anxiety management, rather than the underlying issue itself [69]. Greater inquiry into athletes with EXD could conceivably reveal additional psychopathologies as the source of the compulsion that leads to using exercise for an ameliorative effect [71]. In our view, this is an area that requires detailed scholarly attention.

Equally, overtraining syndrome (OTS) is a prevalent issue for UEAs and gravely affects multiple body systems due to an excess of training without adequate recovery [72]. OTS is characterized by a dysfunction of the hypothalamic–pituitary–adrenal (HPA) axis; this has also been associated with various mental disorders, including depression and suicide [73]. Extreme endocrinological disruption, which is characteristic of OTS, poses a substantial health threat and is implicated in accompanying hormonal, immunological, neurological, gastrointestinal, and musculoskeletal symptoms [74]. Concerningly, endocrine dysfunction can occur whenever there is an imbalance in athlete behaviors around training, recovery, and nutrition. Insufficient recovery from training efforts and/or under fueling to support energy needs causes global hormone-network disruption [75]. This includes the HPA, the reproductive axis, the thyroid axis, and the growth-hormone axis [75]. The duration of unbalanced athlete behaviors determines the extent of endocrine disruption and subsequent adverse impacts on physical and mental health [75]. For the authors, it is imperative that this risk be acknowledged since some recommendations for mental-health management in UEAs advocate for continued physical activity [1]. Future research could investigate whether strengthening the role of sports psychiatry and psychotherapy in the diagnosis of UEAs could be feasible, which might facilitate the prevention or at least the early detection of OTS. From a forensic–psychiatric perspective, professionals could help this community navigate medico-legal interactions, such as anti-doping regulations.

EDs present a considerable concern in this population, and based on our review, may indicate the presence of other serious health concerns, such as low bone-mineral density [43,59], amenorrhea [43], low energy availability [52], and caloric deficit [43]. ED, disordered eating, and low BMI are associated with Relative Energy Deficiency in Sport (RED-S) and may indicate additional risk for hormonal disruption and bone-mass losses in men and women [76]. It is likely that UES-related stereotypes have an effect, as study participants indicated that they believed EDs to be normal and expected in this community [44,57]. These adverse sociocultural notions can render frequently occurring yet abnormal conditions, such as functional hypothalamic amenorrhea, unworthy of detailed investigation [75]. Concerningly, two thirds of the female cyclists in one of the reviewed studies reported being advised to lose weight to improve performance [57].

### 4.3. The Need for Increased Awareness, Scholarly Inquiry, and Regulatory Emphasis 

Based on our assessment, we advocate for future research that examines the UEA population, including recreational and elite athletes. We believe that studies should be designed to understand the prevalence of mental disorders and gender-based trends, aiming to make recommendations for the recognition of potential psychiatric issues in a clinical setting, as well as any physiological comorbidities that may co-occur. Neurobiological research could focus on the discovery of neurotransmitter alteration as a result of high-volume training and the implications for human behavior, since the reviewed literature shows evidence that polymorphisms associated with anxiety and alcohol use may be prevalent in this population [47]. Per our review, the onset of mental disorder is not well understood, and it is unknown whether UEAs are motivated to participate in UESs for amelioration of existing symptoms or whether mental disorders develop from chronic high-volume training. Therefore, in the authors’ opinion, more work should be conducted to discover whether and when the mental-health benefits become annulled with the addition of training volume. 

We believe that information about psychiatric conditions in UEAs could be presented to governing bodies of UESs for regulatory purposes, given the potentially large community affected and the concomitant harmful mental-health effects. UESs are heterogeneous, currently outside the purview of an Olympic committee, and may involve multiple stakeholders. Consequently, this may mean that holistic initiatives are difficult to implement, particularly for those athletes who train independently despite their high motivation and volume of training, or for those who may not otherwise have a direct pathway to mental healthcare. Nonetheless, relevant organizations, like The International Association of Ultrarunners, World Ultracycling Association, Union Cycliste Internationale, and World Triathlon, should be encouraged to create formalized statements on mental health for their respective ultra-endurance communities [77,78,79,80]. Materials for UEAs could be produced to educate individuals on how to avoid any decrement in wellness that would result. 

Finally, as has proven the case in other sporting frameworks—for instance, with Michael Phelps in Olympic swimming—the most impactful messaging often comes from the athletes themselves [81]. We therefore hope that more UEAs are encouraged to speak openly about their mental health and advocate for increased awareness in both media publications and on social media. Tentative steps have been made in this direction by renowned ultra-endurance athletes, such as Cody Beals and Sarah Sturm [82,83]. Greater attention to these concerns could lessen stigma, as well as yield more opportunities for recognition, diagnosis, and adequate treatment in UEAs. 

### 4.4. Limitations

We considered our review strategy to be a suitable methodology to examine evidence of psychiatric disorders in the UEA population. To fully scrutinize attendant issues, more original data are required in sports psychiatry; we hope this paper forms the starting point for primary investigations into this underrepresented population, particularly into prevalence rates and gender-based trends.

Nonetheless, our review has its limitations. Given the restricted knowledge base, the heterogeneity of this community, and the semantic difficulties in providing comprehensive definitions of UESs, we adopted a narrative-review strategy rather than a systematic approach. Narrative reviews can invoke criticism about reproducibility and selection decisions, which cannot be neglected in our methodology [84]; nonetheless, previous literature reviews about mental health in sports have adopted narrative-based methodologies [85]. By only searching across Scopus and PubMed, we may have overlooked studies in more specialized databases. Similarly, it is possible that by using ICD-11 criteria, non-classified mental-health conditions could have been omitted. However, we believe that our review provides a starting basis for additional research in this area and captures general evidence about mental disorders in the UEA community. 

Furthermore, narrative reviews can also elicit concerns about bias [86], which again cannot be discounted in our search process. For example, we excluded papers that did not meet our criteria for UEA athletes as outlined in the introduction. Equally, we did not include articles that specifically investigated how contextual factors like COVID-19 and intra-race conditions affected the mental health of UEAs. Nevertheless, we believed this was appropriate, as our review solely aims to gather data about the intrinsic psychiatric profile of UEAs removed from variables that accompany race situations, such as competitive stress, sleep deprivation, and environmental conditions. Future reviews and empirical research could investigate these specific considerations. 

By excluding a timeframe in our methodology, it is possible that outdated information was included in our review; this was done with the objective of capturing older, critical studies [87], especially given that there is limited evidence in this area. Other reviews in sports psychiatry have used similar approaches [88]. Many of the studies we captured do not incorporate epidemiological comparisons with the general population. As such, existing literature may contain a predisposition for correlational rather than causational relationships between mental illness and UEAs. This again highlights the need for further research in this area, as we have discussed throughout this paper. A more rigorous evidence base would support appropriate regulatory initiatives and advance the goal of protecting athlete well-being.

## 5. Conclusions

We conducted a narrative review of literature on mental disorder in UEAs. Although there are limited available data, results show various psychiatric concerns in this population per ICD-11 diagnostic categories. As there is an abundance of research on the physiological implications of high-volume training associated with UESs, complementary evidence into the mental-health consequences will provide a more holistic view of the overall well-being of UEAs. It is important that healthcare practitioners and the UEA community not conflate athletic ability with health, as we believe that physiology and psychology must be equally considered. Therefore, we advocate for increased inquiry into the psychiatric profiles of UEAs. 

Specifically, more research is needed to uncover the complex relationship between substance addiction and exercise addiction, as well as the potentially dangerous behavioral patterns that may emerge because of neurotransmitter alterations. Educational materials should be made available to athletes, coaches, and healthcare practitioners, which can be informed by a larger evidence base. Requisite regulatory bodies may play a crucial role in developing position statements on mental health that are equal in importance to those on physical health. The popularity of ultra-endurance events is ever-increasing, and we believe that supporting the mental health of this community should match this pace.

## Figures and Tables

**Table 1:** Summary of Reviewed Studies.

Reference	Year	Sample Size	Relevant ICD-11 Classifications	Description of Findings
Morgan et al. [14]	1987	*n* = 400	Depression	Summary of 10-year study of endurance swimmers evaluated for mood using Profile of Mood States (POMS) over a 6-month training block. In all years, mood decreased during the highest-volume training period. A total of 80% were diagnosed with clinical depression.
Morgan et al. [15]	1988	*n* = 12	Depression	Swimmers assessed daily with POMS during a 12-day high-volume block. As training volume increased, sense of well-being and mood decreased, depression increased.
DeBate and Wethington [38]	2001	*n* = 583	Eating disorder	Triathletes assessed for disordered eating using Eating Attitudes Test-26 (EAT-26). A total of 28% of females, 11% males at risk for eating disorder (ED). Evidence of preoccupation with food/weight, calorie control. A total of 100% of participants express body dissatisfaction using Body Test Material.
Blaydon and Lindner [39]	2002	*n* = 203	Eating disorder	Triathletes assessed for EXD and ED using Exercise Dependence Questionnaire (EDQ) and EAT. A total of 30.6% of cohort at risk for primary EXD, 21.6% at risk for secondary EXD, 12.3% at risk for ED.
Harrison et al. [40]	2003	*n* = 33	Depression	Study designed to evaluate the effect of triathlon race on depression using Beck Depression Inventory II. A total of 21% of participants were diagnosed with, and 12% in treatment for, mood disorders. Depression score not associated with mood during race.
Kiraly and Joy [41]	2003	*n* = 1	Depression, psychosis	Case study of male Ironman triathlete with ED, depression, and psychosis.
Yates et al. [42]	2003	*n* = 190	Eating disorder, anxiety disorder, fear-related disorder	Ultra-endurance runners, cyclists, and paddlers examined for ED using Exercise Orientation Questionnaire (EOQ) and self-report of psychiatric symptoms. Risk of ED found in 12% runners, 14% cyclists, and 18% paddlers. Self-loathing was associated with ED symptoms for the entire group (*F*(1) = 4.8, *p* < 0.05) and for females specifically (*F*(1) = 9.30, *p* < 0.001). More anxiety/panic symptoms were found in paddlers (*χ*2 = 7.91, *p* < 0.01). Paddlers had higher self-loathing scores than cyclists (*F*(2) = 6.91, *p* < 0.01). The most anxiety/panic symptoms were reported by the female-only paddler group (*χ*2(1) = 10.27, *p* < 0.001) than the other groups.
Millet and Groslambert [18]	2005	*n* = 4	Anxiety disorder	Elite triathletes evaluated for fatigue and anxiety using a questionnaire unique to the study over a 40-week training block. Increased training loads were correlated to higher anxiety and fatigue.
Hoch et al. [43]	2007	*n* = 15	Eating disorder	Study of disordered eating measured by EAT-26 and bone-mineral density measured by dual-energy x-ray absorptiometry in club triathletes. A total of 60% in caloric deficit, 40% with history of amenorrhea.
Riebl et al. [44]	2007	*n* = 61	Eating disorder	Male cyclists were assessed for ED using EAT-26 and Survey of Eating Disorder Among Cyclists (SEDAC). Risk of ED found in 19.7%. A total of 45.9% of cyclists believed ED to be common in cycling.
Axelsen [45]	2009	*n* = 1	Eating disorder	Autoethnographic case report of one female triathlete with anorexia nervosa.
Magee et al. [46]	2016	*n* = 345	Eating disorder, depression	Study designed to reveal correlations between exercise dependence (EXD), eating patterns as measured by the Three Factor Eating Questionnaire (TFEQ), and psychological distress/depression symptoms as measured by the Kessler 6 Scale in Ironman triathletes. Exercise Dependence Scale (EDS) used to find 30% participants at risk and symptomatic of EXD.
Sanhuenza et al. [47]	2016	*n* = 192	Anxiety disorder, alcohol-use disorder	Triathletes were assessed by psychiatric interview (MINI international neuropsychiatric interview, 5.0), genotyping analysis of ACE rs1799752 (I/D), and serotonin transporter 5HTT (5-HTTLPR). Anxiety disorder was a risk for 44.3% of total participants and 57% of Inferior Performance (IP) group. IP showed higher frequency of polymorphisms for 5-HTTLPR, which is associated with increased anxiety; 5HT1AR-1019C > G, which is associated with anxiety disorder; and NK1R r56715729, which is associated with stress homeostasis, alcohol dependence, and alcohol abuse.
Cook et al. [48]	2017	*n* = 179	Eating disorder	Study of male cyclists used EDS, Drive for Thinness, and Leisure Time Exercise Questionnaire to find that 8.24% were at risk and 70% were symptomatic for primary exercise dependence. A total of 1.18% were at risk and 10.59% were symptomatic for secondary exercise dependence, suggesting presence of ED. EXD more prevalent with more frequent workouts.
Mayolas et al. [49]	2017	*n* = 859	Anxiety disorder, depression	Amateur endurance cyclists assessed for exercise addiction using Exercise Addiction Inventory (EAI), Quality of Life (QoL), and anxiety and depression (Hospital Anxiety and Depression Scale). Evidence of EA found in 17% of cyclists. Lower QoL, assessed by Short Form Survey version 2.0–12, was observed in the at-risk group. EA not related to training volume.
Buck et al. [50]	2018	*n* = 98	Depression	Ultramarathoners assessed for exercise addiction and depression using EAI and PHQ-2. A total of 20% of athletes were positive for EA risk and 20% were positive for depression risk.
Schüler et al. [51]	2018	*n* = 323	Anxiety disorder	Study designed to assess predictors of exercise addiction in ultra endurance athletes using Balanced Measure of Psychological Needs Scale (BMPN), the anxiety-oriented self-control subscale of the Volitional Components Inventory, and EAI. Overall sample scored high on EAI with the mean score at cut-off for EA.
Torstveit et al. [52]	2019	*n* = 53	Eating disorder	Exercise dependence, measured by EDS, correlated with higher training volume in male cyclists and triathletes. Those at risk for EXD were also at risk for ED, measured by the ED Examination Questionnaire (EDE-Q), and showed evidence of low energy availability.
Hauck et al. [53]	2020	*n* = 1022	Eating disorder	In a study of amateur endurance athletes, the prevalence of food addiction, measured by the Yale Food Addiction Scale 2.0, was 6.2%. Prevalence of ED, measured by the Eating Disorder Diagnostic Scale (EDDS), was 6.5%. Prevalence of exercise addiction, measured by the Questionnaire to Diagnose Exercise Dependence in Endurance Sports (FESA), was 30.5%. Results unrelated to the number of training hours per week.
Muros et al. [54]	2020	*n* = 4037	Eating disorder	Study of endurance cyclists and triathletes found 17.9% at risk for ED measured by the revised restraint scale (RRS), SCOFF questionnaire, and Mediterranean Diet Adherence Screener (MEDAS); higher in females and cyclists, lower in males and triathletes.
Bueno-Antequera et al. [55]	2022	*n* = 330	Alcohol-use disorder	Amateur endurance cyclists assessed for exercise addiction using EAI, QoL, using Health Survey 2.0. EXD risk found in 64% of males and 60% females. Lower mental QoL and increased alcohol use was found in 14.8% of participants.
Cook and Dobbin [56]	2022	*n* = 36	Eating disorder	Male cyclists assessed for ED risk using the Sports Nutrition Knowledge Questionnaire (SNKQ) and Brief Eating Disorder in Athletes Questionnaire (BEDA-Q). Risk for ED was high in 11% of group and negatively correlated with understanding of nutrition (*r* = −0.55, *p* = 0.006).
Koppenburg et al. [57]	2022	*n* = 122	Eating disorder	Female cyclists were assessed for ED risk (EAT-26). A total of 32% were found to be at risk and 80% percent of those surveyed believed cycling to be associated with high ED risk.
Colledge et al. [58]	2022	*n* = 123	Depression	High-performance athletes were assessed for EXD using the EXD Scale, evidence of childhood trauma using the Childhood Trauma Questionnaire (CTQ), ADHD using the Homburger ADHS Skalen für Erwachsene (HASE), and depression using the BDI. EXD risk found in 23.6% and those with EXD had higher likelihood of depression (*t*(121) = 4.944, *p* < 0.001), ADHD (*t*(121) = 2.915, *p* = 0.004), and childhood trauma (*t*(121) = 2.297, *p* = 0.024).
Høeg et al. [59]	2022	*n* = 123	Eating disorder	Ultramarathoners assessed using proprietary survey consisting of questions from Female Athlete Triad Screening Questionnaire and Eating Disorder Examination Questionnaire. Risk of ED found in 44.5% of males and 63.5% females. Evidence of bone-stress injury (BSI) found in 37.5% of females and 20.5% of males using dual-energy X-ray absorptiometry (DXA).

## Data Availability

Not applicable.
